# AdipoRon, an Orally Active, Synthetic Agonist of AdipoR1 and AdipoR2 Receptors Has Gastroprotective Effect in Experimentally Induced Gastric Ulcers in Mice

**DOI:** 10.3390/molecules26102946

**Published:** 2021-05-15

**Authors:** Hubert Zatorski, Maciej Salaga, Marta Zielińska, Kinga Majchrzak, Agata Binienda, Radzisław Kordek, Ewa Małecka-Panas, Jakub Fichna

**Affiliations:** 1 Department of Biochemistry, Medical University of Lodz, 92-215 Lodz, Poland; hubert.zatorski@umed.lodz.pl (H.Z.); salaga.maciej@gmail.com (M.S.); zielinska.martt@gmail.com (M.Z.); kinga3m@op.pl (K.M.); agata.binienda@gmail.com (A.B.); 2 Department of Digestive Tract Diseases, Medical University of Lodz, 93-281 Lodz, Poland; ewa.malecka-panas@umed.lodz.pl; 3 Department of Pathology, Medical University of Lodz, 92-215 Lodz, Poland; radzislaw.kordek@umed.lodz.pl; 4 Department of Biochemistry, Faculty of Medicine, Medical University of Lodz, Mazowiecka 6/8, 92-215 Lodz, Poland

**Keywords:** AdipoRon, adiponectin receptors, gastroprotection, oxidative stress

## Abstract

Introduction: Adiponectin is a hormone secreted by adipocytes, which exhibits insulin-sensitizing and anti-inflammatory properties and acts through adiponectin receptors: AdipoR1 and AdipoR2. The aim of the study was to evaluate whether activation of adiponectin receptors AdipoR1 and AdipoR2 with an orally active agonist AdipoRon has gastroprotective effect and to investigate the possible underlying mechanism. Methods: We used two well-established mouse models of gastric ulcer (GU) induced by oral administration of EtOH (80% solution in water) or diclofenac (30 mg/kg, p.o.). Gastroprotective effect of AdipoRon (dose 5 and 50 mg/kg p.o.) was compared to omeprazole (20 mg/kg p.o.) or 5% DMSO solution (control). Clinical parameters of gastroprotection were assessed using macroscopic (gastric lesion area) and microscopic (evaluation of the gastric mucosa damage) scoring. To establish the molecular mechanism, we measured: myeloperoxidase (MPO), superoxide dismutase (SOD), catalase (CAT), and glutathione peroxidase (GPX) activities; glutathione (GSH) level; and IL-1β, adenosine monophosphate-activated protein kinase (AMPK), and phosphorylated AMPK expression in gastric tissue. Results: AdipoRon produced a gastroprotective effect in both GU mouse models as evidenced by significantly lower macroscopic and microscopic damage scores. AdipoRon exhibited anti-inflammatory effect by reduction in MPO activity and IL-1β expression in the gastric tissue. Moreover, AdipoRon induced antioxidative action, as demonstrated with higher GSH levels, and increased SOD and GPX activity. Conclusions: Activation of AdipoR1 and AdipoR2 using AdipoRon reduced gastric lesions and enhanced cell response to oxidative stress. Our data suggest that AdipoR1 and AdipoR2 activation may be an attractive therapeutic strategy to inhibit development of gastric ulcers.

## 1. Introduction

Gastric ulcers (GU) are considered as one of the most prevalent gastrointestinal (GI) tract disorders worldwide. GU are characterized predominantly by an imbalance in acid and pepsin production and inappropriate mucosal response, which leads to gastric tissue injury [1]. Multiple additional factors have been connected with the development of gastric lesions, such as functional alterations in mucus-bicarbonate layer and gastric mucosal blood flow. Moreover, a significant decrease in superoxide dismutase (SOD) and glutathione peroxidase (GPX) activities were reported in marginal mucosa of human gastric ulcers and experimental animals [2,3]. Furthermore, neutrophils, which are one of the major endogenous sources of free radicals, infiltrate into gastric mucosa of humans or animals infected with *H. pylori* [4,5].

The most common causes of GU are *Helicobacter pylori* and the use of non-steroidal anti-inflammatory drugs (NSAIDs). However, GU not caused by *H. pylori* infection has nowadays become an important issue [6]. Recent research found that obesity and increased visceral adiposity was associated with an increased risk of GU, in particular *H. pylori*-negative ulcers [6,7,8]. Visceral fat was recently recognized as an endocrine organ that secretes a vast number of biologically active substances, such as leptin, adiponectin, visfatin, resistin, and tumor necrosis factor-α (TNF-α). Adiponectin is one of the most investigated bioactive products of the adipose tissue, with a proven anti-diabetic, anti-inflammatory, and anti-atherogenic effects, and it acts by AdipoR1 and AdipoR2 receptors [9]. AdipoR1 and AdipoR2 expression was significantly higher in peripheral blood of patients with gastric cancer compared to healthy subjects [10]. Furthermore, expression of AdipoR1 in gastric cancer cells assessed by immunohistochemical staining was associated with significantly longer survival rates in patients with gastric cancer in comparison to patients from negative staining group. Moreover, in the same study, Tsukada et al. showed that adiponectin presents antiproliferative effect in gastric cancer cell lines (MKN45 and NUGC3) suggesting that adiponectin has the possibility to be involved in cell growth suppression through AdipoR1 [11].

Adiponectin and adiponectin receptor expression was confirmed in majority of tissues in the human body, including the stomach [10,12,13]. In a study performed by Kentish et al., adiponectin mRNA was detected in the mouse gastric tissue, suggesting that adiponectin is locally produced in the murine stomach [14]. The presence of adiponectin protein in the gastric mucosa was confirmed by Kentish et al. in gastric antrum sections. Moreover, adiponectin-producing cells were found to be co-localized with 5-HT, gastric intrinsic factor, gastrin, and histamine indicating that adiponectin is produced in enterochromaffin cells, chief cells, G-cells, and enterochromaffin-like cells, respectively [14]. Furthermore, AdipoR1 and AdipoR2 mRNA was identified in gastric mucosal and muscular neuron cell bodies in mice [14]. Data obtained by Idrizaj et al. support this observation by showing, using touchdown-PCR analysis, AdipoR1 and AdipoR2 expression in mouse gastric fundus [15].

While adiponectin is mainly produced in the adipose tissue, its anti-inflammatory effects may be observed also in the GI tract. Consequently, in a study performed by Sideri et al. AdipoR1 silencing using intracolonic administration of siRNA in mice caused exacerbation of trinitrobenzene sulfonic acid (TNBS) colitis [16].

Moreover, the adiponectin and its receptors play role in development of different cancers [17]. Effect of adiponectin seems to be mediated, at least in part, by an increase in fatty-acid oxidation via activation of adenosine monophosphate-activated protein kinase (AMPK) and also via peroxisome proliferator-activated receptor (PPAR)-α [18,19,20]. Interestingly, serum adiponectin levels are decreased in obese subjects and this leads to metabolic disorders [17]. Moreover, recent studies showed that low serum levels of adiponectin are associated, independently of BMI, with increased risk of developing gastritis in humans [17].

Despite the therapeutic potential of adiponectin, its clinical use has significant disadvantages, including the high probability of adverse immunoreactions, the requirement of high dosage and constant intravenous (i.v.) injection to elicit beneficial effects, and the challenges associated with producing adiponectin protein on a large scale [21].

An adiponectin receptor agonist, AdipoRon was recently developed by Okada-Iwabu et al. [22]. This synthetic orally active small molecule binds to and activates both AdipoR1 and AdipoR2, ameliorates insulin resistance and type 2 diabetes, and prolongs the shortened lifespan of db/db mice. Current research shows that AdipoRon may be a novel therapeutic molecule that effectively treats diabetes, but also has cardioprotective properties [22,23]. However, whether AdipoRon may possess gastroprotective properties, attenuating inflammatory reactions in gastric tissue and enhancing anti-oxidative stress defense mechanisms have not been previously investigated.

In this study, we tested the hypothesis whether an AdipoR1 and AdipoR2 receptor agonist, AdipoRon, has gastroprotective effect in mouse models of gastric lesions. We used two well-established models, EtOH- and NSAID-induced GU. Clinical parameters for gastroprotection were assessed based on the gastric lesion area and the number of GU. In order to establish the mechanism of gastroprotective action, an inflammatory cytokine: IL-1β as well as oxidative stress defense mechanisms, such as catalase (CAT), SOD, and GPX activity and glutathione (GSH) levels were quantified in gastric tissues. Moreover, the impact of AdipoRon administration on expression of pAMPK/AMPK in the mouse gastric tissue was investigated.

## 2. Results

### 2.1. AdipoRon Exhibits Gastroprotective Effect in the EtOH-Induced Gastric Ulcer Model in Mice

**Figure 1:**
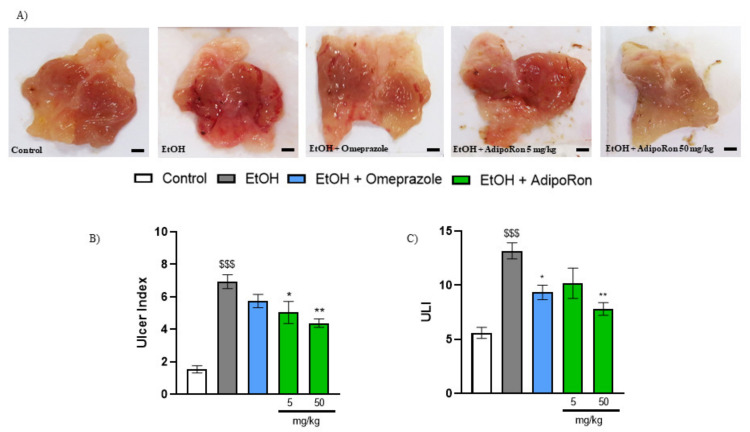
Representative images of the mouse stomach from (**A**) control, EtOH, EtOH + omeprazole 20 mg/kg, EtOH + AdipoRon 5 mg/kg, and EtOH + AdipoRon 50 mg/kg and ulcer index (**B**) and ULI (**C**). Significant differences were observed after administration of AdipoRon at the dose of 50 mg/kg p.o. $$$ *p* < 0.001, as compared to control mice, * *p* < 0.05, ** *p* < 0.01, as compared to EtOH-group. Scale bar: 1 mm. Results are expressed as mean ± SEM for n = 5–6 mice per group.

To investigate the gastroprotective effect of AdipoRon, we used the mouse EtOH-induced model of GU. As shown in Figure 1, administration of 80% solution of EtOH resulted in reproducible gastric lesions, manifested by an increased ulcer index and ULI compared with control. Omeprazole which was used as a reference drug and administered orally at the dose of 20 mg/kg p.o. significantly attenuated GU development as shown by decreased ulcer index and ULI (ulcerative lesion index) (Figure 1). In gross examination, administration of AdipoRon resulted in reduction in gastric lesion area (Figure 1A). Moreover, AdipoRon reduced the total ulcer area, as demonstrated by decreased ulcer index, and total number of ulcers, as demonstrated by decreased ULI in a dose-dependent manner. AdipoRon at the dose of 5 and 50 mg/kg p.o. resulted in similar reduction in ulcer index and ULI as compared to the reference drug, omeprazole.

**Figure 2:**
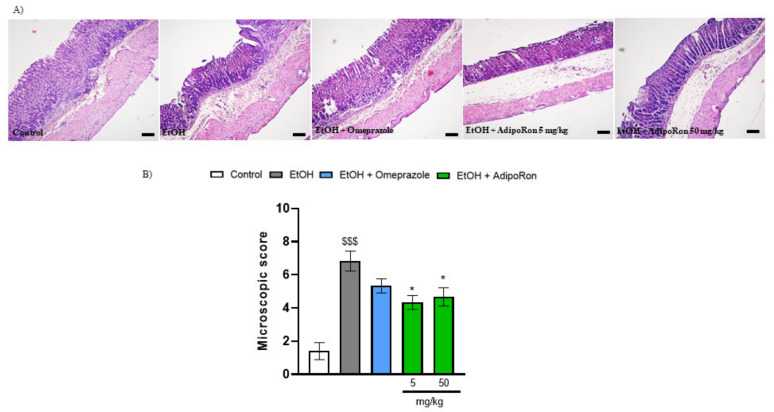
Representative micrographs of hematoxylin and eosin-stained sections of the mouse stomach from (**A**) control, EtOH, EtOH + omeprazole 20 mg/kg, EtOH + AdipoRon 5 mg/kg, and EtOH + AdipoRon 50 mg/kg; (**B**) histogram showing the microscopic score for each experimental group. AdipoRon at doses 5 and 50 mg/kg significantly reduced gastric mucosa damage as shown by decrease in the microscopic score. $$$ *p* < 0.001, as compared to control mice, and * *p* < 0.05, as compared to EtOH-group. Scale bar: 100 µm and microscope magnification: 40×. Results are expressed as mean ± SEM for n = 5–6 mice per group.

The microscopic evaluation of gastric sections stained with hematoxylin and eosin was in good agreement with observation of macroscopic parameters (Figure 2). Histological analysis of stomach sections from untreated animals showed intact epithelium and absence of edema in the upper mucosa (Figure 2A). Severe microscopic damage, characterized by epithelial cell loss, presence of edema in the upper mucosa, and extensive cellular infiltration was observed in gastric specimens after administration of EtOH (Figure 2A). The histological changes were reversed after p.o. administration of omeprazole (Figure 2A). Treatment with AdipoRon in dose 5 and 50 mg/kg alleviated edema in the upper mucosa and reduced cellular infiltration (Figure 2A). Nevertheless, in microscopic evaluation, the dose-dependent effect was not observed (Figure 2B,C). Microscopic evaluation showed that AdipoRon may be considered as a more potent gastroprotective drug compared to omeprazole (Figure 2). Based on gathered information, AdipoRon with dose of 50 mg/kg was chosen for further experiments.

### 2.2. AdipoRon Has Anti-Inflammatory Effect in the EtOH-Induced Gastric Ulcer Model in Mice

**Figure 3:**
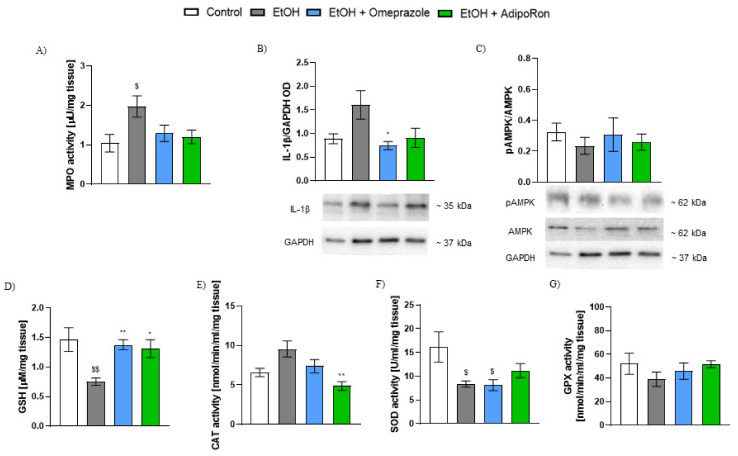
Effect of AdipoRon on inflammatory markers and anti-oxidant mechanisms in the mouse stomach in EtOH-induced gastric ulcer model. Figure shows changes in (**A**) MPO activity, (**B**) IL-1β levels, (**C**) pAMPK/AMPK ratio, (**D**) GSH, (**E**) CAT, (**F**) SOD activity, and (**G**) GPX activity. AdipoRon at the dose of 50 mg/kg p.o. significantly reduced MPO activity, IL-1β expression, and CAT activity. Moreover, AdipoRon increased GSH levels and SOD activity. $ *p* < 0.05 and $$ *p* < 0.01, as compared to control mice, and * *p* < 0.05 and ** *p* < 0.01 as compared to EtOH-group. Results are expressed as mean ± SEM for n = 5–6 mice per group.

As shown in Figure 3, administration of 80% solution of EtOH resulted in reproducible inflammation is gastric tissue, manifested by significantly increased MPO activity and non-significantly increased IL-1β levels. The MPO activity and IL-1β levels after administration of omeprazole as well as AdipoRon (50 mg/kg p.o.) were non-significantly reduced compared to EtOH-treated group (Figure 3A,B).

Moreover, treatment with AdipoRon increased the pAMPK/AMPK ratio in the gastric tissue, but results did not reach statistical significance (Figure 3C).

### 2.3. AdipoRon Enhances the Antioxidant Mechanisms in the Mouse Stomach in the EtOH-Induced Gastric Ulcer Model

Administration of EtOH impaired the antioxidant mechanism in gastric tissue as demonstrated by significant decrease in GSH levels, increase in CAT activity, and diminished SOD activity as compared to control (Figure 3D–F). 

AdipoRon, as well as omeprazole administration resulted in a significant increase in GSH levels in comparison to EtOH-treated group (Figure 3D). Moreover, AdipoRon administration reduced the CAT and increased SOD activity in the EtOH-induced gastric ulcer model (Figure 3E,F). The antioxidant effect was not present in the omeprazole-treated group.

There were no significant differences observed in the GPX activity in all experiment groups (Figure 3G).

### 2.4. AdipoRon Has Gastroprotective Effect in the NSAID-Induced Gastric Ulcer Model

**Figure 4:**
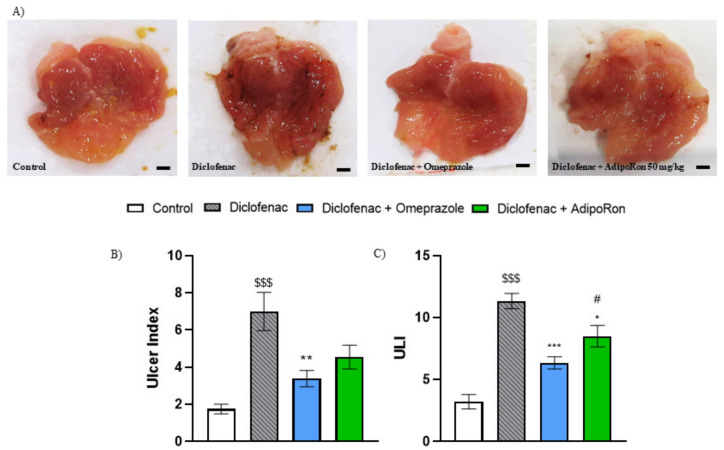
Macroscopic score and representative images of the mouse stomach from (**A**) control, diclofenac 30 mg/kg, diclofenac + omeprazole 20 mg/kg, and diclofenac + AdipoRon 50 mg/kg. AdipoRon exerted gastroprotective effect in the mouse model of diclofenac-induced gastric ulcer as shown by a decrease in the ulcer index (**B**) and ULI (**C**). $$$ *p* < 0.001, as compared to control mice; * *p* < 0.05, ** *p* < 0.01 and *** *p* < 0.001, as compared to diclofenac group; and # *p* < 0.05, as compared to omeprazole group. Scale bar: 1 mm. Results are expressed as mean ± SEM for n = 5–6 mice per group.

To confirm the results obtained with EtOH-induced model, we used NSAID-induced GU model. As shown in Figure 4, administration of diclofenac (30 mg/kg p.o.) resulted in reproducible gastric lesions, manifested by an increased ulcer index and ULI. Macroscopic examination showed that administration of AdipoRon (50 mg/kg p.o.) resulted in reduction in gastric lesion area, i.e., decreased the ulcer index and ULI, however the statistical significance was observed only in ULI. Furthermore, in NSAID-induced gastric ulcer model AdipoRon exhibited weaker gastroprotective effect compared to omeprazole as evidenced by a statistically significant difference in ULI compared to the reference drug (Figure 4C).

**Figure 5:**
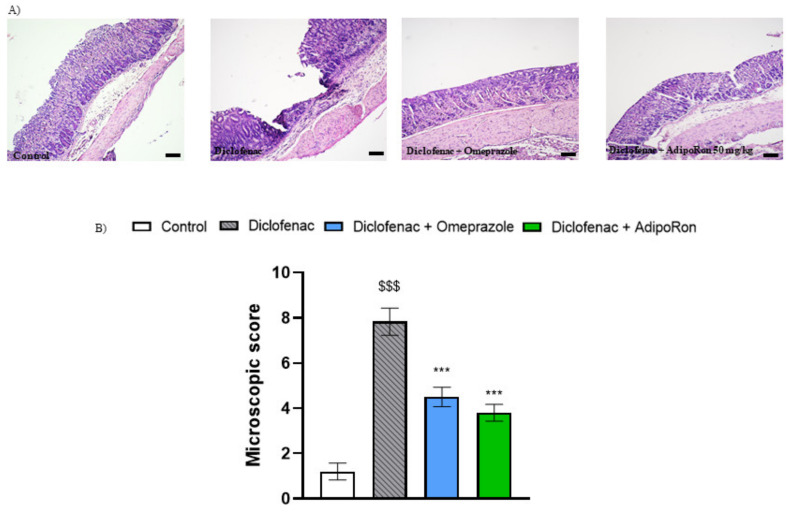
Representative micrographs of hematoxylin- and eosin-stained sections of the mouse stomach from (**A**) control, diclofenac 30 mg/kg, diclofenac + omeprazole 20 mg/kg, and diclofenac + AdipoRon 50 mg/kg; (**B**) histogram showing the microscopic score of each experimental group. AdipoRon significantly reduced mucosal injury as shown by a decrease in the microscopic index. $$$ *p* < 0.001, as compared to control mice, and *** *p* < 0.001, as compared to diclofenac group. Scale bar: 100 µm and microscope magnification: 40×. Results are expressed as mean ± SEM for n = 5–6 mice per group.

Histological analysis of stomach sections from untreated animals showed intact epithelium and absence of edema in the upper mucosa (Figure 5). The microscopic evaluation of gastric sections stained with hematoxylin and eosin revealed severe microscopic damage in the NSAID-treated group, characterized by epithelial cell loss, presence of edema in the upper mucosa and infiltration of immune cells. Gastric tissue damage was attenuated after p.o. administration of omeprazole as well as p.o. administration of AdipoRon, as evidenced by reduction in the microscopic score.

### 2.5. AdipoRon Has Anti-Inflammatory Effect in the NSAID-Induced Gastric Ulcer Model

**Figure 6:**
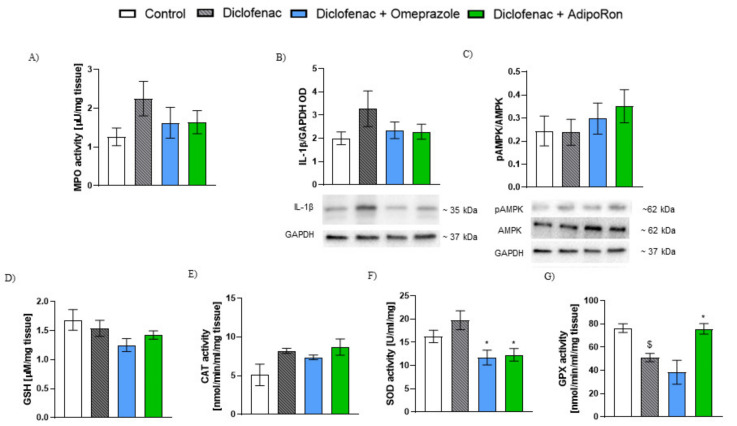
Effect of AdipoRon on inflammatory markers and antioxidant mechanisms in the mouse stomach in diclofenac-induced gastric ulcer model. Figure shows changes in (**A**) MPO activity, (**B**) IL-1β levels, (**C**) pAMPK/AMPK ratio, (**D**) GSH, (**E**) catalase, (**F**) SOD, and (**G**) GPX activity. AdipoRon 50 mg/kg p.o. reduced MPO activity, IL-1β expression, and SOD activity and increased GPX activity. AdipoRon had no effect on GSH levels and CAT activity in diclofenac induced model of gastric ulcer in mice. $ *p* < 0.05, as compared to control mice, and * *p* < 0.05 as compared to diclofenac-group. Results are expressed as mean ± SEM for n = 5–6 mice per group.

Administration of diclofenac resulted in a non-significant increase in MPO activity and IL-1β levels in gastric tissue (Figure 6A,B). Activation of AdipoR1 and AdipoR2 by AdipoRon resulted in a decrease in MPO activity and IL-1β level, however, differences did not reach statistical significance (Figure 6A,B). Moreover, administration of AdipoRon insignificantly increased the pAMPK/AMPK ratio in the mouse gastric tissue (Figure 6C).

### 2.6. AdipoRon Enhances the Antioxidant Mechanisms in the Mouse Stomach in the NSAID-Induced Gastric Ulcer Model

Administration of diclofenac resulted in a non-significant increase in CAT and SOD activity and a significant decrease in GPX activity in the mouse gastric tissue (Figure 6 E–G). There were no differences in the level of GSH in all experimental groups (Figure 6D). AdipoRon administration slightly increased CAT activity in the gastric tissue and significantly decreased SOD activity (Figure 6E,F). Furthermore, administration of AdipoRon resulted in a significant increase in GPX activity compared to diclofenac-treated group (Figure 6G).

## 3. Discussion

GU is one of the most prevalent disorders of the GI tract, characterized by an imbalanced immunological reaction that leads to development of stomach lesions. Aro et al. unexpectedly found that 25% of gastric ulcers and 19% of duodenal ulcers were *H. pylori*-negative [24]. Yamamoto et al. [8] evaluated whether lower serum adiponectin level is associated with the risk of endoscopic gastritis. In their study, in which 2400 participants were enrolled, serum adiponectin levels were significantly lower in patients with gastritis compared to subjects without gastritis. Furthermore, multivariate logistic regression analysis revealed that lower serum adiponectin level (OR 0.96; 95% CI 0.93–0.99) were significantly associated with endoscopic erosive gastritis [8].

Adiponectin is an adipocyte-derived cytokine acting through AdipoR1 and AdipoR2 [25]. While these receptors contain seven transmembrane domains, they are topologically distinct from G protein-coupled receptors (GPCR) [25]. In consequence, while most adipokines, e.g., TNF-α are proinflammatory cytokines, adiponectin reduces oxidative/nitrative stress, protects cells from apoptosis, inhibits leukocyte-endothelial interaction, and decreases smooth muscle proliferation [26]. 

The gastroprotective effect of the AdipoR1 and AdipoR2 agonist, AdipoRon, in the GI tract has not been investigated before. Previous studies focused on adiponectin itself or used adiponectin-knockout mice [27,28]. Yamamoto et al. [28], using adiponectin-knockout mice, demonstrated that a deficiency in adiponectin exacerbated gastric lesions induced in mice by oral administration of EtOH. In another study, performed by Fart et al. [27] gastroprotective effect of adiponectin in EtOH-induced GU model was evaluated in rats. Dose-dependent anti-ulcerogenic effect after intraperitoneal injection of adiponectin was observed. Additionally, reduced leucocyte infiltration was observed in microscopic evaluation.

Although numerous studies demonstrated that adiponectin supplementation exerts anti-inflammatory, antidiabetic, and antiatherosclerosis properties, adiponectin application is limited due to multiple factors such route of administration and high cost of production [21]. Moreover, current pharmacological approach to GU treatment is based on drugs targeting gastric acid production such as proton pump inhibitors and H2-receptors antagonists. These drugs, while often effective, are not completely free of adverse effects. Taken together aforementioned, there is an urgent need to develop new therapeutics that exert anti-inflammatory and antioxidant effects in gastric tissue.

In our study, we tested the hypothesis that gastric lesions are alleviated by activation of AdipoR1 and AdipoR2. Furthermore, we also aimed to unravel the mechanism that may be involved in this anti-ulcer effect. We demonstrated that AdipoR1 and AdipoR2 activation led to a gastroprotective effect in well-established mouse models of GU, as evidenced by reduction in the ulcer index and ULI. Moreover, we showed that administration of AdipoRon resulted in attenuation of inflammatory state at molecular levels, as evidenced by reduction in MPO activity, an important indicator of inflammation seen in ulcer lesions and related to extensive neutrophil infiltration and aggregation in gastric tissue [29], and a decrease in the expression of inflammatory cytokine IL-1β. Additionally, we demonstrated that activation of AdipoR1 and AdipoR2 receptors develops its gastroprotective effect via an anti-oxidative pathway in the GI tract. Hence, AdipoRon may be a promising therapeutic tool in the treatment of GU. 

Omeprazole is an antisecretory drug inhibiting parietal cell H+/K+ ATP pump, which plays a role in the final step of gastric acid production [30]. In turn, omeprazole suppresses gastric basal and stimulated acid secretion and its effect occurs rapidly within 1 h of administration [31]. Due to its established properties, omeprazole is widely used as a reference drug in animal models of gastric ulcer to evaluate the efficiency of potential new therapeutics. In the EtOH-induced gastric ulcer model, administration of AdipoRon at the dose of 5 and 50 mg/kg resulted in comparable to omeprazole gastroprotective effect. Moreover, AdipoRon administration resulted in a decrease in inflammatory markers, such as MPO activity and IL-1β expression in the manner similar to the reference drug. In turn, in the NSAID-induced gastric ulcer model, administration of AdipoRon resulted in a significantly higher ULI compared to omeprazole, whereas ulcer index and microscopic score were comparable. In addition, AdipoRon resulted in a similar non-significant reduction in evaluated inflammatory markers as omeprazole. The observed differences in the effectiveness of AdipoRon and omeprazole may be explained by the characteristics of used mouse models of gastric ulcers. EtOH, once administered, causes membrane damage, cell exfoliation, and erosion. Moreover, ROS formation, reduction in prostaglandin synthesis and increased synthesis of leukotrienes is observed. Thus, the use of EtOH-induced gastric ulcer model allows the induction of gastric ulcer by direct action on the mucosa. In turn, NSAIDs are suggested to induce gastric lesions through inhibition of prostaglandin synthesis, neutrophil accumulation, reduction in blood flow, and reduction in mucosal cell proliferation as well as ROS production

Oxidative stress, depletion of antioxidants, neutrophil accumulation, increased numbers of inflammatory cytokines, and reduced blood supply to the gastric mucosa have all been shown to be implicated in the pathophysiology of GU [1,32]. Exposure to harmful agents leads to excessive production of reactive oxygen species (ROS), which are causing gastric mucosa injury. In turn, the mucus layer and endogenous antioxidants, which are part of the GI defense system, are crucial in protecting against ROS-induced cytotoxicity [33]. ROS such as superoxide, H_2_O_2_, and hydroxyl radicals cause tissue damage, whereas oxygen-handling cells contain antioxidant enzymes, such as SOD, CAT, and GPX, able to protect them against the toxic effects of ROS [34,35].

Our results indicate that activation of AdipoR1 and AdipoR2 stimulates anti-ROS defense mechanism in the stomach attenuating the gastric damage induced by both EtOH and high dose NSAIDs in different manners. Administration of AdipoRon in EtOH-induced model resulted in the increase in GSH levels and SOD activity and reduction in CAT activity. In turn, oral gavage of AdipoRon in NSAID-induced GU model resulted in decrease in SOD activity and increase in GPX activity, with no effect on CAT activity and GSH levels. This observation suggests that activation of AdipoR1 and AdipoR2 may result in stimulation of different protection mechanism against exogenous oxidants and irritants. Such attribute could be exploited in the treatment and/or prevention of upper GI disorders.

To date, mechanism explaining the gastroprotective properties of AdipoR1 and AdipoR2 activation is not fully understood. Yamamoto et al. [28] in their study showed that gastroprotective role of adiponectin may be partially mediated by stimulation of wound repair through increased expression of prostaglandin E2 (PGE2). Fart et al. suggested that adiponectin effect in gastric motility and production of mucus may be responsible for gastroprotective effect [27]. Nevertheless, studies investigating the anti-inflammatory properties of AdipoRon in other organs may suggest other possible explanations.

In the study by Zhang et al., the effect of AdipoRon on myocardial ischemia/reperfusion injury was evaluated [23]. Administration of AdipoRon in dose 50 mg/kg resulted in improved cardiac function, attenuated post-ischemic cardiomyocyte apoptosis and significantly alleviated postischemic oxidative stress, as evidenced by reduced nicotinamide adenine dinucleotide phosphate (NADPH) oxidative expression and superoxide production [23]. Importantly, AdipoRon attenuated postischemic myocardial apoptosis through both AMPK-mediated and AMPK-independent signaling. In another study, Jenke et al. [36] showed that AdipoRon reduces inflammation and impairment of cardiac function associated to systemic inflammatory response syndrome induced by cardiopulmonary bypass (CPB) in rats. Oral gavage of AdipoRon resulted in activation of AMPK and reduction in CPB-upregulation of TNF-α and IL-β as well as NADPH oxidase and inducible nitric oxide synthase (iNOS) [36]. Gu et al. [37] demonstrated that AdipoRon protects against contrast-induced nephropathy by suppressing oxidative stress and inflammation by activation of the AMPK pathway. Administration of AdipoRon in dose 50 mg/kg significantly reversed serum creatinine, blood urea nitrogen, and creatinine clearance induced by iopromide in Sprague–Dawley rats. AdipoRon administration resulted in decrease in MPO activity and IL-1β expression levels as well as restored SOD activity and GSH levels [37].

Importantly, majority of the studies on AdipoRon showed that the AMPK pathway seemed to be the leading pathway in its physiological functions [38,39]. Activation of the AMPK pathway plays important roles in regulating oxidation and inflammation [40]. Here, we investigated the involvement of AMPK in gastroprotective effects of AdipoRon in the GU mice model induced by EtOH and NSAIDs. In line with the abovementioned studies, treatment with AdipoRon resulted in an increased ratio of pAMPK/AMPK proving that the AMPK pathway is involved in the protective effects of AdipoRon on GU model.

## 4. Materials and Methods

### 4.1. Animals

Male balbC mice (weight: 22–24 g), obtained from the Animal House of the University of Lodz, Poland, were used for all experiments. Animals were maintained under a 12-h light/dark cycle and housed at a constant temperature (22–23 °C) in sawdust-lined plastic cages with free access to chow pellets and tap water ad libitum. The study was carried out in accordance with the recommendations described in the Guide for the Care and Use of Laboratory Animals of the Medical University of Lodz, Poland. All experiments on animals were approved by the Local Ethical Committee for Animal Experiments (Protocol #23/ŁB170/2020). Groups of 5–6 animals were used in all in vivo experiments. Efforts were made to minimize animal suffering and to reduce the number of animals used. All in vivo experiments have been performed in accordance with ARRIVE guidelines (for details please see [41]).

### 4.2. EtOH-Induced Ulcer Model

The experiment was performed according to the method described by Robert et al. [42] with some modifications. Mice were randomly divided into groups and fasted for 12 h before experiment but had free access to water. One hour before administration of ulcerogenic agent, mice were orally treated with a single dose of vehicle, omeprazole, or AdipoRon. All mice, apart from control group, received 80% solution of EtOH to induce gastric lesions. The concentration of EtOH used in this study was determined based on previous experiments [1,32]. After 30 min the animals were sacrificed and the stomachs were removed, opened along greater curvature, gently rinsed with PBS to remove gastric content and blood clots, and photographed. The quantification of the gastric mucosa damage induced by ethanol was performed in blinded manner using ImageJ (National Institutes of Health, Washington DC, USA) to calculate following parameters: (1) the ulcer index expressed as a percentage of ulcerated area in relation to the area of the stomach corpus and (2) ulcerative lesion index (ULI). For calculation of ULI, ulcers were classified as level I, ulcer area <1 mm^2^; level II, ulcer area 1–3 mm^2^; and level III, ulcer area >3 mm^2^. ULI was determined as 1 × (number of ulcers level I) + 2 × (number of ulcers level II) + 3 × (number of ulcers level III) [43].

### 4.3. Diclofenac-Induced Ulcer Model

The experiment was performed according to the method described by Shimoyama et al. [44] with some modifications. After 12 h fasting, mice were randomly divided into groups of 5–6 animals. One hour before administration of ulcerogenic agent, mice were orally treated with a single dose of vehicle, omeprazole, or AdipoRon. All mice, but not control group, received diclofenac to induce gastric lesions. After 4 h, animals were sacrificed. The process of gastric tissue isolation and evaluation of the damage was the same as procedures used in EtOH-induced ulcer model.

### 4.4. Pharmacological Treatment

EtOH, diclofenac, omeprazole, and AdipoRon were dissolved in dimethyl sulfoxide (DMSO) and further diluted with saline to a final DMSO concentration of 5%. The solution of 5% DMSO in saline alone constituted a vehicle for control groups and had no impact on the observed parameters. AdipoRon was administered per os (p.o.) at doses of 5 and 50 mg/kg. Omeprazole was administered p.o. at dose of 20 mg/kg and diclofenac was administered p.o. at the dose 30 mg/kg. All reagents were purchased from Sigma-Aldrich (Poznan, Poland) unless otherwise stated. AdipoRon (5 and 50 mg/kg), diclofenac (30 mg/kg), and omeprazole (20 mg/kg) was dissolved in 5% DMSO. Authors have chosen the dose of AdipoRon based on available data. Original study showed that AdipoRon binds to both AdipoR1 and AdipoR2 in vitro (Kd1.8 and 3.1 µM, respectively) and activates AMPK. When AdipoRon was administered orally to mice (50 mg/kg), it was confirmed that the concentrations of AdipoRon in plasma (Cmax of 11.8 µM) reached levels greater than the Kd values (AdipoR1, 1.8 µM; AdipoR2, 3.1 µM) [22]. Moreover, it has been shown that plasma AdipoRon reaches the maximum concentration at 2 h after oral gavage. In a study performed by Chun-Laam Ng et al. in the mouse model of Alzheimer’s disease, AdipoRon reached the peak (16,419.9 ng/mL) at 2 h after oral administration of dose (50 mg/kg) [31]. Furthermore, in the aforementioned study, it has been shown that phosphorylation of AMPK was significantly increased 1 h after AdipoRon administration. Thus, 50 mg/kg of AdipoRon was chosen as dose for further studies. 

### 4.5. Histology

After obtaining macroscopic images, segments of the gastric tissue were stapled flat, mucosal side up, into cardboard, and fixed in 10% neutral-buffered formalin for 24 h at 4 °C. Samples were then dehydrated, embedded in paraffin, sectioned at 5 µm, and mounted into slides. Next, sections were stained with hematoxylin and eosin and examined using microscope Zeiss Axio Imager setup (Zeiss, Jena, Germany). A qualified observed performed all the histopathologic procedures in blinded manner to avoid any bias. A microscopic total damage was determined in a 0–14 scale according to the criteria described by Laine and Weinstein [45] similarly to our previous work [1]. Briefly, each histological section was examined for epithelial cell loss (0–3), edema in the upper mucosa (0–4), hemorrhagic damage (0–4), and the presence of inflammatory cells (0–3).

### 4.6. Determination of Tissue MPO Activity

In order to monitor the degree of inflammation, MPO activity was determined using a standardized method, as described earlier [46]. Briefly, stomach segments (approximately 15 mg) were homogenized in hexadecyltrimethylammonium bromide (HTAB) buffer (0.5% HTAB in 50 mM potassium phosphate buffer, pH 6.0; 50 mg tissue/mL). Homogenates were centrifuged (15 min, 13,200× *g*, 4 °C). On a 96-well plate, 200 μL of 50 mM potassium phosphate buffer (pH 6.0), supplemented with 0.167 mg/mL of O-dianisidine hydrochloride and 0.05 μL of 1% hydrogen peroxide, was added to 7 μL of the supernatant. Absorbance was measured at 450 nm after 30 and 60 s (iMARK Microplate Reader, Bio-Rad, Hertfordshire, UK). All measurements were performed in triplicate. MPO activity was expressed in milliunits per gram of wet tissue, 1 unit being the quantity of enzyme able to convert 1 μmol hydrogen peroxide to water in 1 min at room temperature.

### 4.7. Determination of IL-1β, Expression by Western Blotting

Sections of the stomach (10–15 mg) were isolated, washed with PBS, and kept at −80 °C until further analysis. Tissue homogenates were prepared using Mammalian Cell Lysis Kit according to manufacturer’s protocol (cat. no. MCL1-KT, Sigma Aldrich, Poznan, Poland). Concentration of total protein was evaluated in each sample using the Pierce 660 nm protein assay (Thermo Scientific, Rockford, IL, USA) in triplicate. Separation of proteins (15 µg/well) was performed on Mini-PROTEAN^®®^ TGX™ gels (Bio-Rad, Warsaw, Poland) in electrophoretic buffer (0.1% SDS, 192 mM glycine, 25 mM Tris, pH 8.3). Separated proteins were electrotransferred onto PVDF membranes (pore size, 0.45 µm; Life Technologies, Carlsbad, CA, USA) in transfer buffer (20% (*v*/*v*) methanol, 192 mM glycine, 25 mM Tris, pH 8.3). The membranes were incubated for 1 h at room temperature in 5% non-fat dry milk in PBS with Tween 20 (0.1% m/v; PBST) to saturate non-specific protein binding sites. Then, membranes were probed with the following primary antibodies diluted in 1% non-fat dry milk in PBST for 80 min at 25 °C: mouse monoclonal IL-1β (1:1000, sc-32294 1:1000 Santa Cruz Biotechnology, Santa Cruz, CA, USA), rabbit monoclonal AMPK antibody (1:1000, D5A2, Cell Signaling Technology, Massachusetts, USA); rabbit monoclonal pAMPK (1:1000, Thr172, Cell Signaling Technology, Massachusetts, USA), glyceraldehyde-3-phosphate dehydrogenase (GAPDH; 1:15 000; MAB374; Merck Millipore, Warsaw, Poland) as a reference protein. Appropriate horseradish-peroxidase (HRP)-conjugated secondary antibody (1:3000 for IL-1β and 1:6000 for the others) was applied for 1 h at room temperature, and then bands were visualized using Super Signal West Femto Western blotting substrate (Thermo Scientific, Rockford, IL, USA) as a substrate for the localization of HRP activity. Qualitative and quantitative analysis was performed by measuring integrated optical density (IOD) by ImageLab v.5.2.1 for Windows^TM^ program (Bio-Rad SA, Warsaw, Poland). For determination of protein weight, we have used Precision Plus Protein Standards (Bio-Rad SA, Warsaw, Poland).

### 4.8. Determination of SOD Activity

SOD activity in the gastric tissue was determined using Superoxide Dismutase Assay Kit (Cayman Chemicals, Ann Arbor, MI, USA), which utilizes a tetrazolium salt for detection of superoxide radical generated by xanthine oxidase and hypoxanthine. Briefly, tissue samples were homogenized in ice-cold 20 mM HEPES buffer, pH = 7.2, containing 1 mM ethylene glycol-bis(2-aminoethylether)-N,N,N′,N′-tetra acetic acid(EGTA), 210 mM mannitol, and 70 mM sucrose per gram of tissue. Subsequently, homogenates were centrifuged at 1500× *g* for 5 min at 4 °C. Supernatants were collected and underwent the procedure described in the manufacturer’s protocol. Absorbance was measured at 450 nm (iMARK Microplate Reader, Bio-Rad, UK). All measurements were performed in triplicate. SOD activity was expressed in milliunits per gram of wet tissue, with 1 U being the quantity of enzyme needed for 50% dismutation of the superoxide radical at room temperature. Units of SOD activity were calculated from a standard curve using purified bovine erythrocyte SOD enzyme.

### 4.9. Determination of CAT Activity

CAT activity in the gastric tissue was determined using Catalase Assay Kit (Cayman Chemicals, Ann Arbor, MI, USA) The kit utilized the peroxidative function of CAT for determination of its enzymatic activity, and the method is based on the reaction of the enzyme with methanol in the presence of H2O2. Briefly, tissue samples were homogenized in ice-cold buffer containing 50 mM potassium phosphate, pH = 7.0 and 1 mM EDTA per gram of tissue. Homogenates were centrifuged at 10,000× *g* for 15 min at 4 °C. Supernatants were put on ice and underwent the procedure described in the manufacturer’s protocol. Absorbance was measured at 540 nm (iMARK Microplate Reader, Bio-Rad, UK). All measurements were performed in duplicate. CAT activity was expressed in milliunits per gram of wet tissue, with 1 U being the amount of enzyme that causes the formation of 1.0 nmol of formaldehyde per minute at room temperature. Units of CAT activity were calculated from standard curve using purified bovine liver CAT enzyme. 

### 4.10. Determination of GPX Activity

GPX activity in the gastric tissue was determined using Glutathione Peroxidase Assay Kit (Cayman Chemicals, Ann Arbor, MI, USA), which measures indirectly GPX activity by a coupled reaction with glutathione reductase (GR). Oxidized glutathione (GSSG), produced upon reduction in hydroperoxide by GPX, is recycled to its reduced state by GR and NADPH. The oxidation of NADPH to NADP+ is accompanied by a decrease in absorbance at 340 nm. Briefly, tissue samples were homogenized in cold 50 mM Tris-HCl buffer, pH = 7.5, containing 5 mM EDTA and 1 mM dithiothreitol (DTT) per gram of tissue. Subsequently, homogenates were centrifuged at 10,000× *g* for 15 min at 4 °C. Supernatants were collected and underwent the procedure described in the manufacturer’s protocol. Absorbance was measured at 340 nm (iMARK Microplate Reader, Bio-Rad, UK). All measurements were performed in duplicated. GPX activity was expressed in milliunits per gram of wet tissue, with 1 U defined as the amount of enzyme that will cause the oxidation of 1.0 nmol of NADPH to NADP+ per minute at 25 °C.

### 4.11. Measurement of Reduced GSH Levels

Glutathione levels in the gastric tissue were determined using Glutathione Assay Kit (Cayman Chemicals, Ann Arbor, MI, USA), which utilizes an enzymatic recycling method, using glutathione reductase, for the quantification of GSH. The sulfhydryl group of GSH reacts with DTNB (5,5′-dithio-bis-2-(nitrobenzoic acid), Ellman’s reagent) and produces a yellow colored 5-thio-2-nitrobenzoic acid (TNB). The mixed disulfide, GSTNB (between GSH and TNB) that is concomitantly produced, is reduced by GR to recycle the GSH and produce more TNB. The rate of TNB production is directly proportional to this recycling reaction which is in turn directly proportional to the concentration of GSH in the sample. Shortly, tissue samples were homogenized in cold 50 mM 2-(N-morpholino)-ethanesulfonic acid (MES) buffer, pH = 7.0 containing 1 mM EDTA per gram of tissue. Subsequently, homogenates were centrifuged at 10,000× g for 15 min at 4 °C. Supernatants were collected and underwent the procedure described in the manufacturer’s protocol. Absorbance was measured at 410 nm (iMARK Microplate Reader, Bio-Rad, UK) after 25 min. All measurements were performed in duplicated. GSH concertation was read off a standard curve and expressed as µM of GSH per milligram of wet tissue.

### 4.12. Statistics

Statistical analysis was performed using Prism 5.0 (GraphPad Software Inc., La Jolla, CA, USA). Data are expressed as means ± SEM. Shapiro–Wilk normality test was performed. Next, one-way ANOVA followed by Tukey post hoc tests was used for analysis. *p* values < 0.05 were considered statistically significant.

## 5. Conclusions

Nowadays, obesity is one of the major healthcare problems worldwide. Importantly, obesity—through increased production of selected adipokines, e.g., leptin and TNF-α—is associated with low-grade inflammation. In contrast, adiponectin, which is one of the most abundant adipokines in the human body, possesses anti-inflammatory actions and its levels are reduced in obese people. This study showed for the first time that AdipoR1 and AdipoR2 activation by AdipoRon ameliorated GU formation by alleviation of inflammation and enhancement of the antioxidant defense mechanism in the stomach in two different mouse models of GU. Furthermore, this study showed that the effect of AdipoRon was mediated through AMPK signaling pathway. Thus, we suggest that the activation of AdipoR1 and AdipoR2 receptors may be beneficial in the gastric lesion therapy. Nevertheless, further pre-clinical studies are needed to evaluate the potential involvement of other signaling pathways, such as ERK 1/2 and Akt, as well human studies to investigate the safety profile and efficiency of adiponectin therapy. 

## Data Availability

The data presented in this study are available on request from the corresponding author.

## Sample Availability

Not available.
